# Reassessing the role of progesterone receptor isoforms PGR-A and PGR-B in female fertility

**DOI:** 10.1126/sciadv.adz7757

**Published:** 2026-08-12

**Authors:** Doan T. Dinh, Kirsten M. Smith, Tim McPhee, Natalie J. Foot, David C. Bersten, Melissa A. White, Paul Q. Thomas, Rebecca L. Robker, Darryl L. Russell

**Affiliations:** ^1^ School of Pharmacy and Biomedical Sciences, and Robinson Research Institute, Adelaide University, Adelaide, Australia.; ^2^ South Australian Health and Medical Research Institute, Adelaide, Australia.

## Abstract

Progesterone is a critical reproductive hormone that acts via progesterone receptor transcriptional regulators. The short PGR-A isoform lacks a 164–amino acid amino-terminal region present in PGR-B that markedly enhances transcriptional activity. Isoform-specific mutants inactivating either PGR-A (PRAKO) or PGR-B (PRBKO) indicated that PGR-A is specifically essential for female fertility. This study revises that interpretation by showing that an inadvertent frameshift mutation in the PRAKO caused ablation of both isoforms, not the intended PGR-A isoform–specific mutation. A true PGR-A–specific mutant generated through CRISPR-Cas9 editing with a complete lack of PGR-A but retained PGR-B expression showed a phenotype indistinguishable from wild type. Thus, while the short PGR-A isoform has distinct function, it is not independently essential for female reproduction in mice, and the physiological role of these highly conserved isoforms must be reconsidered in light of this new information.

## INTRODUCTION

**Fig. 1.**
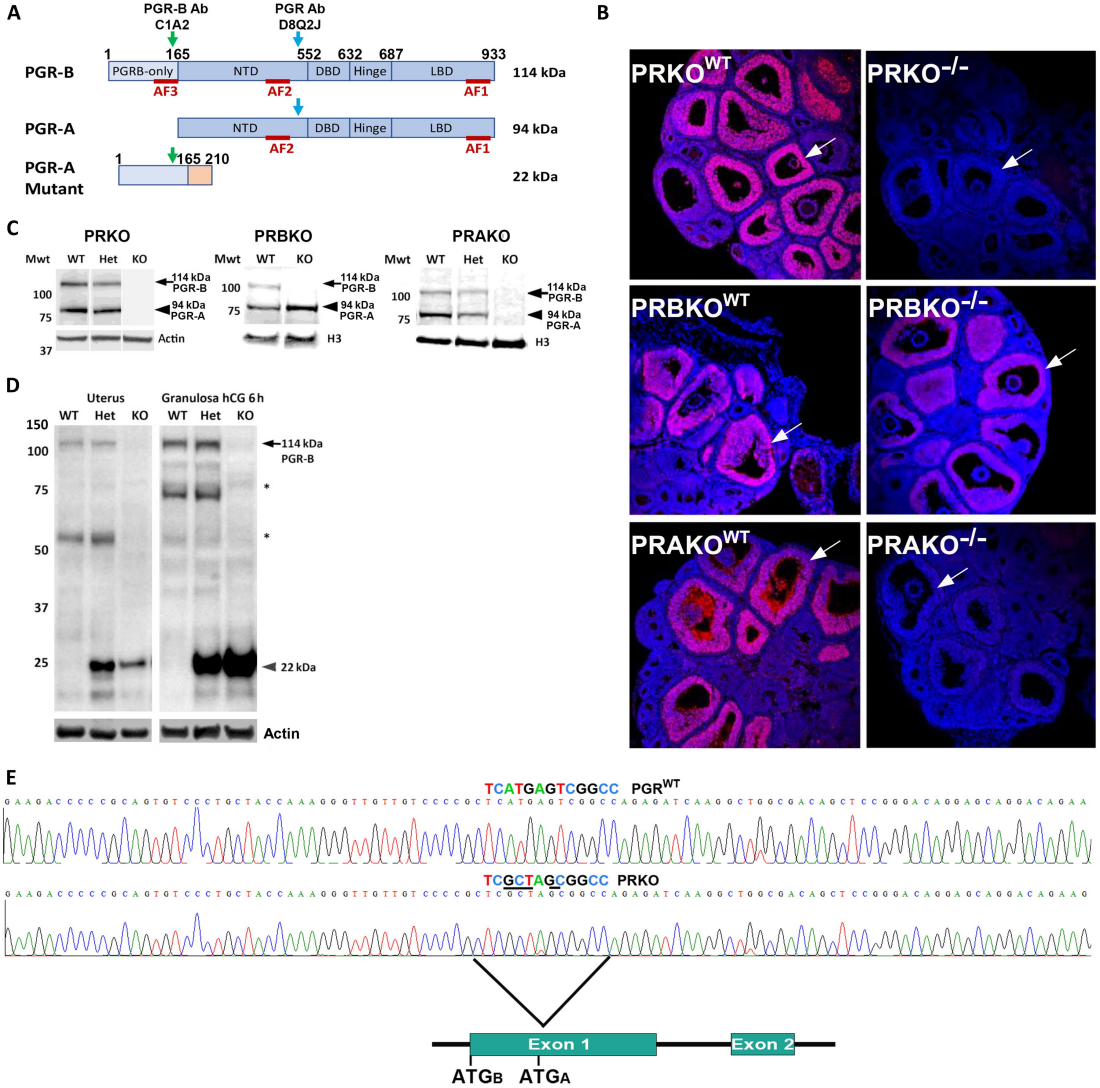
PGR protein in PRKO, PRBKO, and PRAKO mouse models. (**A**) Protein structure of PGR-A, PGR-B, and the truncated N-terminal protein fragment identified in PRAKO mutants. Epitopes of the different antibodies used, where known, are indicated by arrows. Activation function regions are denoted AF1, AF2, and AF3. The orange region indicates 45 out-of-frame amino acid residues from the frameshift mutation to the first stop codon. Ab, antibody. (**B**) Immunofluorescence with hPRa7 antibody (pan-PGR; epitope undefined) in 6-hour hCG-treated mouse ovaries from WT or homozygous mutants of the PRKO, PRAKO, or PRBKO mouse lines. White arrows indicate examples of preovulatory follicles in each panel. (**C**) Western blot with D8Q2J antibody in uterus extracts from PRKO, PRAKO, or PRBKO of indicated genotype. Positions of molecular weight markers (Mwt) shown on left, Arrows indicate 11-kDa PGR-B, and arrowheads indicate 94-kDa PGR-A. (**D**) Western blot of uterus or ovarian granulosa cells (pooled from two animals of each genotype) from the PRAKO mouse model [WT, heterozygous (Het), or knockout (KO)] using the PGR-B N-terminal domain antibody, C1A2. Arrow indicates 114-kDa full-length PGR-B, and arrowhead indicates 22-kDa fragment of the PGR-B N-terminal domain. Asterisks indicate 55-kDa uterus and 65-kDa granulosa cell products that are unidentified. β-Actin or H3 loading control as indicated. h, hours. (**E**) Sanger sequencing comparison of the PGR-A translation start site in genomic DNA from WT (PGRWT) or the original PGR-A knockout (PRAKO) mice. Mutated ATG start site and the point deletion are indicated by underlined regions.

Progesterone receptor (PGR) is a hormone-responsive transcription factor produced as two major isoforms, PGR-A and PGR-B coexpressed in all progesterone responsive tissues, including ovulating ovaries (*1*). These are alternatively translated isoforms originating from two alternative start sites in the first exon. Both isoforms contain the ligand-binding domain and DNA binding domain required for transcriptional activity, while the longer PGR-B isoform includes an additional 164 N-terminal amino acids containing the activation function-3 domain that interacts with cofactors, giving it additional transcriptional activation capability (Fig. 1). PGR-A has been shown to be a relatively weak activator of transcription or, in many cases, to be an antagonist of PGR-B transactivation (*1*).

At ovulation, the mid-cycle luteinizing hormone surge triggers rapid and pervasive reprogramming of gene expression mediating structural remodeling of the ovarian follicle, release of the cumulus oocyte complex, and luteinization of cells to form the corpus luteum. Transiently induced PGR in granulosa cells of mice (*2*), humans, and other mammalian species (*3*) is the only gene shown to be individually critical for ovulation. In humans, competitive antagonists of PGR, mifepristone and ulipristal acetate, block ovulation (*4*) as emergency contraceptives. PGR gene knockouts in mice have been shown to completely prevent ovulation both in whole body or granulosa cell conditional gene disruption (*2*, *5*), confirming that granulosa cell expression of PGR is a master regulator of ovulation. Upon expression in granulosa cells, PGR mediates transcriptional induction of many genes with roles mediating follicle rupture, but not luteinization. Specifically, *Adamts1*, *Edn2*, and *Pparg* are PGR-dependent genes with confirmed roles in ovulation (*2*, *6*–*8*). Uterine PGR is essential for embryo implantation through induction of critical genes including *Nr2f2*, *Ihh*, and *Hand2* (*1*).

Whether the two PGR isoforms have specific functions in physiological processes, particularly in female fertility, was addressed through mutations of either the PGR-B– or PGR-A–specific ATG translation start sites in two mouse models (*9*, *10*). PGR-B mutant mice showed normal fertility, demonstrating that PGR-B is not essential for ovulation or uterine development and implantation, and only a mild change in mammary gland branching morphogenesis was observed (*10*). However, unexpectedly, disruption of the weaker transcription factor, PGR-A, caused infertility and near complete ovulation failure (*9*). The reproductive deficiency in PRAKO mice has been accepted as evidence for a physiologically relevant PGR-A isoform–specific regulatory mechanism. However, there has been no direct mechanistic explanation of how the shorter PGR-A isoform mediates a unique PGR action. We recently applied RNA sequencing (RNA-seq) in granulosa cells comparing total PRKO, as well as the PGR-A– and PGR-B–selective mutants [PRAKO (*9*) and PRBKO (*10*)], showing highly similar transcriptomes in granulosa cells of total PRKO and PGR-A mutants, while the PGR-B mutant transcriptome was nearly indistinguishable from wild type (WT) (*11*).

In this study, we examined the ovary and uterus of the isoform-specific PGR mutant models to understand PGR-regulated mechanisms necessary for ovulation and implantation. Unexpectedly, we discovered that the “isoform-specific” PRAKO mice (*9*) also fail to express PGR-B, thus lacking both isoforms. To fully confirm the reported PGR-A mutant phenotype, we used CRISPR editing to create a bona fide PGR-A–specific mutant mouse. Unexpectedly, this newly generated PGR-A^mut^ line contradicts the previously published finding (*9*) and overturns the current understanding of PGR isoform action. This profoundly reverses the long-held understanding that PGR-A and PGR-B have distinct roles and the significance for control of reproduction.

## RESULTS

### Both PGR protein isoforms are undetectable in PGR-A mutant mouse model

The anovulation in the PRAKO mice, along with the highly similar differential gene expression in granulosa cells in total PRKOs and PRAKO (*11*), suggested that PGR-A has a specific essential role in inducing ovulatory gene induction. However, Western blot and immunolabeling were unable to detect any PGR-B in the presumptive PGR-A–specific mutant uteri or granulosa cells after 6 hours of human chorionic gonadotropin (hCG) treatment (Fig. 1 and fig. S1). In WT mice from each line, immunostaining with hPRA-7 pan-PGR antibody detected PGR in follicles of periovulatory ovaries, while PGR was not detectable in PRKO or PRAKO mutant ovaries (Fig. 1B). Likewise, Western blot with the D8Q2J antibody detected both PGR-A and PGR-B in uteri of WT mice from each line, and PGR-A was detected in PRBKO mutants; however, neither PGR-A nor PGR-B was detected in uteri of PRAKO mutants (Fig. 1C). Our findings suggested that the disruption of PGR-A translation start codon affected PGR-B protein production. Western blots with the C1A2 antibody specific to the unique PGR-B N-terminal domain identified high abundance of a truncated protein of 22 kDa, corresponding to the predicted PGR-B N-terminal protein truncated shortly after the mutated PGR-A start site in granulosa cells and uterus of heterozygous and homozygous mutant mice from the PRAKO line (Fig. 1D).

Sanger sequencing of the PGR locus uncovered an unexpected single-base deletion immediately adjacent to the mutated ATG site in PRAKO. This second mutation introduced a frameshift resulting in a noncoding transcript with multiple premature stop codons after a short random nucleotide sequence (Fig. 1, A and D). The expected ATG>GCT (methionine to alanine) mutation of the PGR-A–specific start site was identified; however, the additional silent mutation in the neighboring serine codon (AGT>AGC) that was engineered to introduce a NheI restriction enzyme cut site (GCTAGC) for genotyping, instead, had a single-base deletion (AGT>AG-). This caused a frameshift in the coding sequence but retained the NheI restriction site modification because the next base after the deletion remained the C required for NheI digestion (Fig. 1E). As a result, genotyping of these mutants using NheI cleavage, as in (*9*), fails to identify the unintended frameshift mutation. The frameshift introduced a stop codon 45 amino acid residues after the PGR-A start site mutation, truncating protein translation from the PGR-B start site after 210 amino acids encoding the PGR-B N-terminal domain (Fig. 1A). Sequencing of the mutated region in genomic DNA of PRBKO confirmed the expected ATG>CTG codon mutation that ablates PGR-B production and the expected silent CTG>CTC mutation encoding valine was present (not shown). Thus, the PRBKO genome modification was as expected, disrupting PGR-B translation but allowing normal PGR-A translation from the downstream ATG start site as confirmed by Western blots (Fig. 1C). These findings confirmed the unexpected protein data, leading to the conclusion that the PRAKO are null for all PGR isoforms.

### Generation of true PGR-A isoform–specific mutant mice

**Fig. 2.**
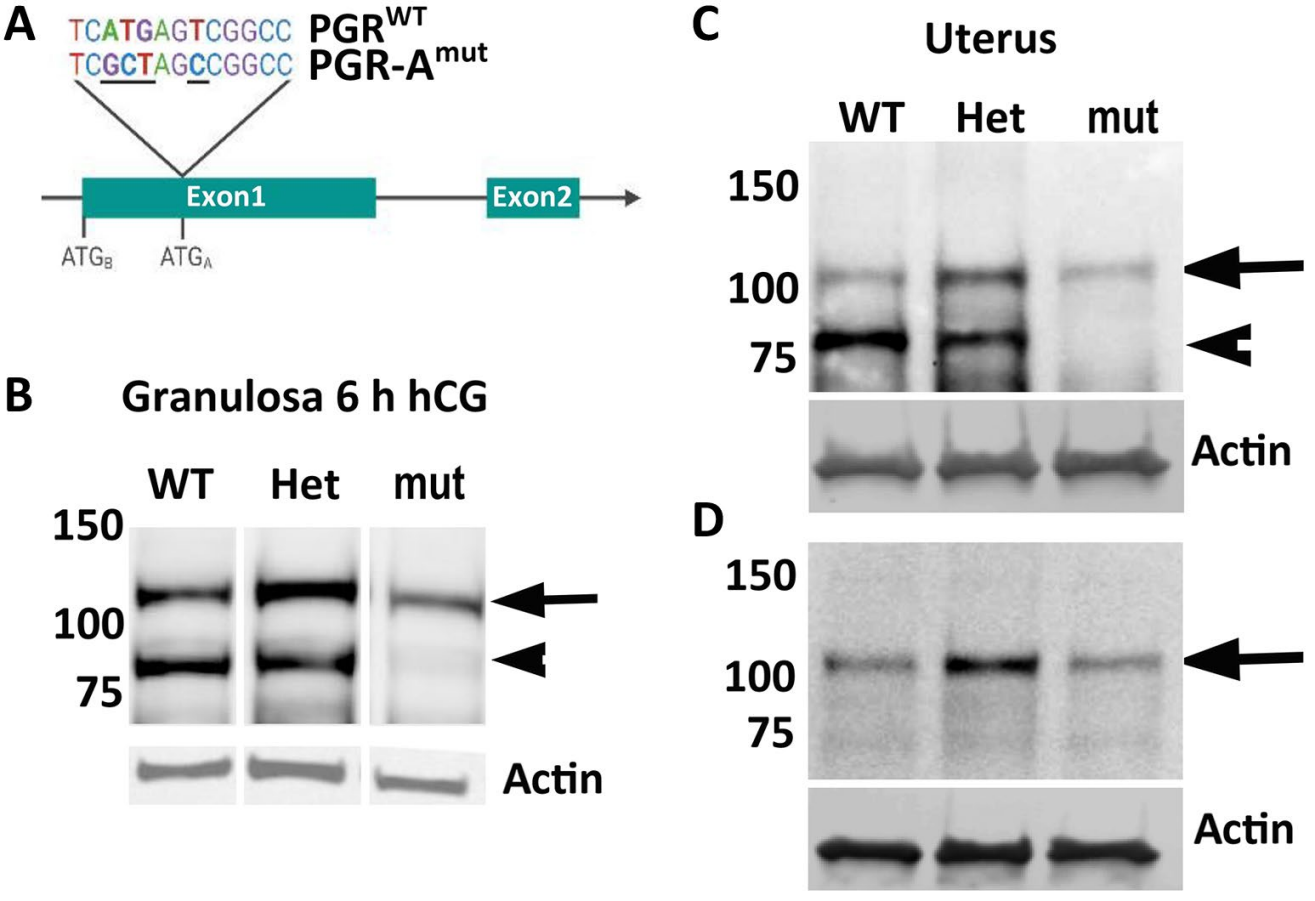
Design and validation of PGR-A–specific mutant mice (PGR-A^mut^). (**A**) Mutant sequence that disrupts the PGR-A–specific ATG start codon to GCT, and an additional T>C silent mutation to engineer the Nhe1 restriction site for genotyping. Altered nucleotides are underlined. (**B**) Western blot of granulosa cell extracts from 6-hour hCG-treated mice using D8Q2J (pan-PGR) antibody. h, hours. (**C**) Western blot of uterus extracts using D8Q2J antibody. (**D**) Western blot of uterus extracts using C1A2, PGR-B–specific antibody. Arrow, 114-kDa PGR-B; arrowhead, 94-kDa PGR-A.

To elucidate the true PGR-A mutant reproductive phenotype and whether PGR-A has specific roles in female fertility, we generated a new mutant mouse model to test unambiguously whether PGR-A is essential for ovulation, pregnancy, and mammary function. Microinjection of zygotes with Cas9 mRNA, PGR-A–targeting pegRNA, and a single-stranded DNA repair template encoding the desired PGR mutation, followed by embryo transfer, produced 36 offspring born from seven surrogate females. Genotyping by NheI digestion of a polymerase chain reaction (PCR) product targeting the PGR-A start site identified 14 offspring with introduced NheI restriction sites. Sanger sequencing of amplicons from six males with NheI–positive digests identified three males with the intended mutation and no other changes to the PGR alleles (Fig. 2A). These founders were bred to WT females, establishing three separate mutant colonies that were independently investigated.

Heterozygous F_1_ offspring from each line were further validated through whole-genome sequencing, demonstrating that progeny from all three founder lines inherit the intended alleles with ATG>GCT mutation and the silent T>C with no other mutations. Heterozygous breeding pairs from each line were established to produce WT, heterozygous, and mutant offspring. The PGR-A start site mutant (PGR-A^mut^) females produced from each line were healthy and viable as expected. The absence of the PGR-A isoform and the presence of the PGR-B isoform were validated by Western blot in uteri and granulosa cells at 6 hours post-hCG using a pan-PGR detecting antibody, which demonstrated successful ablation of PGR-A (Fig. 2, B and C). Heterozygous PGR-A mutants showed reduced levels of the PGR-A isoform (Fig. 2). Likewise, Western blot using the PGR-B–specific antibody also confirmed that PGR-B was present in PGR-A mutant ovaries and uteri, with no truncated fragments identified (Fig. 2D). These data confirm successful and specific deletion of only the PGR-A isoform in the newly generated PGR-A mutant line.

### PGR-A isoform–specific mutant mice ovulate normally

**Fig. 3.**
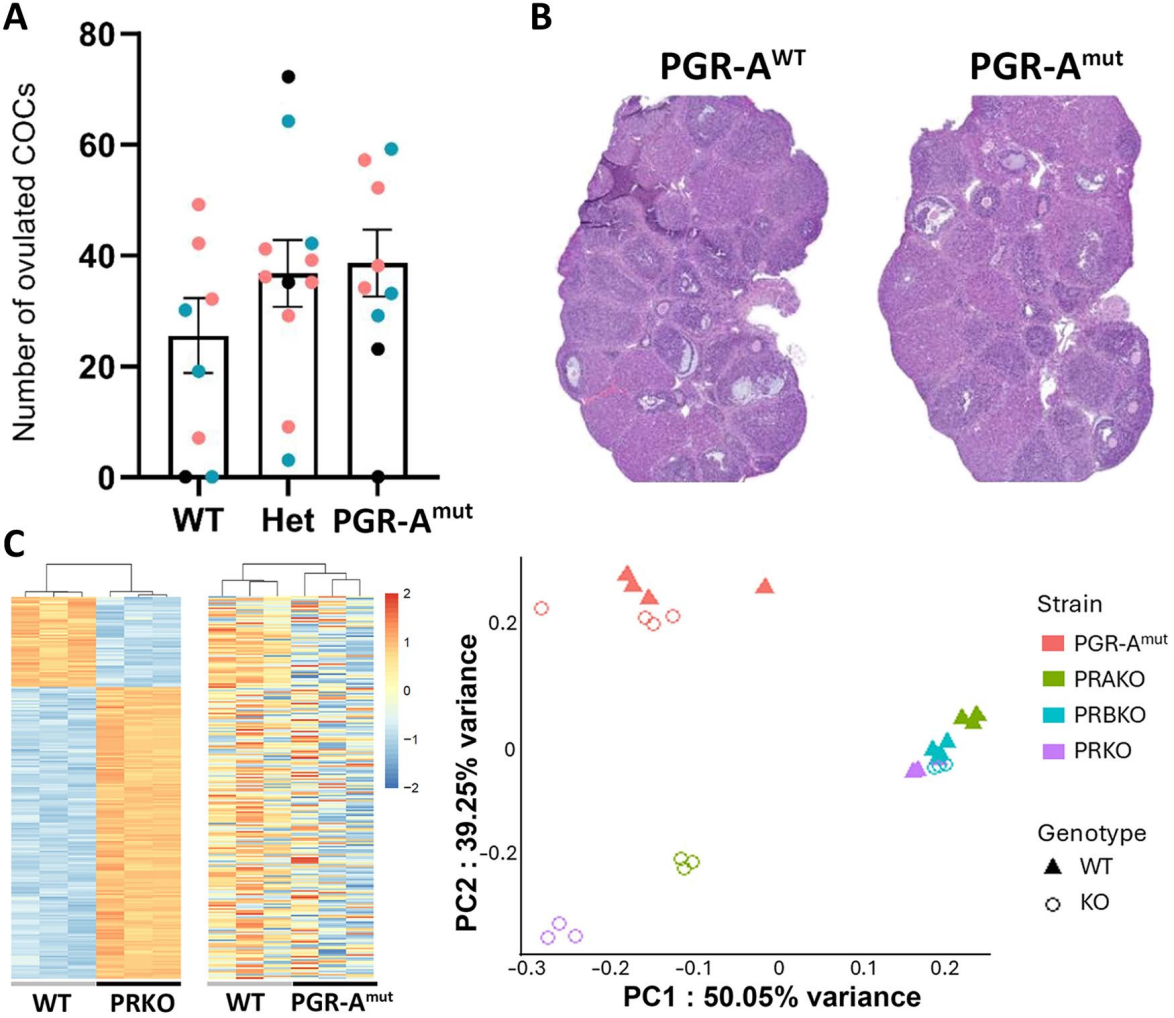
Normal ovarian function in PGR-A^mut^ mice. (**A**) The number of ovulated COCs in oviducts after 16 hours after hCG treatment in mice that were WT, heterozygous (Het) or homozygous for the PGR-A^mut^ mutation. Each color (black, blue, or pink symbols) represents a separate line of founder animals (*n* = 8 to 11 per genotype). (**B**) Histology of representative ovaries from PGR-A^mut^ and WT littermates showing normal follicle and CL numbers and morphology. (**C**) Heatmap showing comparison of DEGs in PRKO and PGR-A^mut^ granulosa cells compared with cells from WT littermates. Principal components analysis of expression from RNA-seq in granulosa cells of WT versus knockout or mutants from each mouse line, PRKO, PRBKO, PRAKO, or the newly generated PGR-A^mut^ line. PC1, principal component 1.

We next performed ovulation assessment in females from each founder line. The number of oocytes ovulated in response to gonadotropin stimulation was similar in homozygous mutant, heterozygous, and WT females (Fig. 3A). The female PGR-A^mut^ mice ovulated an average of 39 cumulus-oocyte complexes (COCs) per mouse, which was not significantly different to PGR^+/+^ and PGR^+/mut^ ovulation rates (22 and 37, respectively). Assessment of animals from each of the three separate founder origins showed consistent results of normal ovulation in PGR-A–specific KO mice. Histological analysis of ovarian sections from these mice following ovulation identified morphologically normal corpora lutea in each genotype (Fig. 3B) with minimal instances of follicles with morphological signs of failure to ovulate such as oocytes trapped within large antral follicles. These data demonstrate that, contrary to previous findings, the PGR-A isoform is not essential for ovulation.

Gene expression profiles regulated by PGR in granulosa cells were assessed by RNA-seq comparison of PGR-A^mut^ with WT littermate females (Fig. 3, D and E, and fig S2). Granulosa cells from PGR-A^mut^ compared with their WT littermates showed very few significant differences in transcripts that were mainly noncoding. Only one known PGR-dependent granulosa cell gene, *Lyve1*, was found to be significantly reduced in PGR-A^mut^ (fig. S2). As we previously reported, significant differences in gene expression in granulosa cells of WT versus PRKO littermates were observed. However, the new PGR-A^mut^ line showed no consistency with the DEG pattern in PRKO granulosa cells (Fig. 3C). This is similar to our previous finding that PRBKO granulosa cells show essentially no differences in the gene expression (*11*). Unsupervised cluster analysis of the PGR-dependent gene set was able to separate the WT from PGR-A^mut^ samples, indicating that subtle differences in global gene expression were detectable. Principal components analysis comparing transcriptomes in granulosa cells of WT versus mutants from PRKO, PRBKO, PRAKO, or the newly generated PGR-A^mut^ line demonstrates that global gene expression profiles of PRKO and PRAKO mice were most closely clustered with each other and separate from their WT littermates. However, gene expression patterns from PGR-A^mut^ or PRBKO^−/−^ were indistinguishable from their WT littermates (Fig. 3C), indicating that only mouse lines with both PGR-A and PGR-B ablation show transcriptome changes compared with their WT controls. The difference in mouse strain resulted in unique clustering of the WT and PGR-A^mut^ expression profile. These results indicate that independent PGR-B– or PGR-A–deficient mice are equally able to promote normal ovulatory gene expression, establish pregnancy, and undergo parturition, with mammary function sufficient to support pup growth.

### Normal uterine and mammary gland morphology and normal female fertility in PGR-A^mut^ mice

**Fig. 4.**
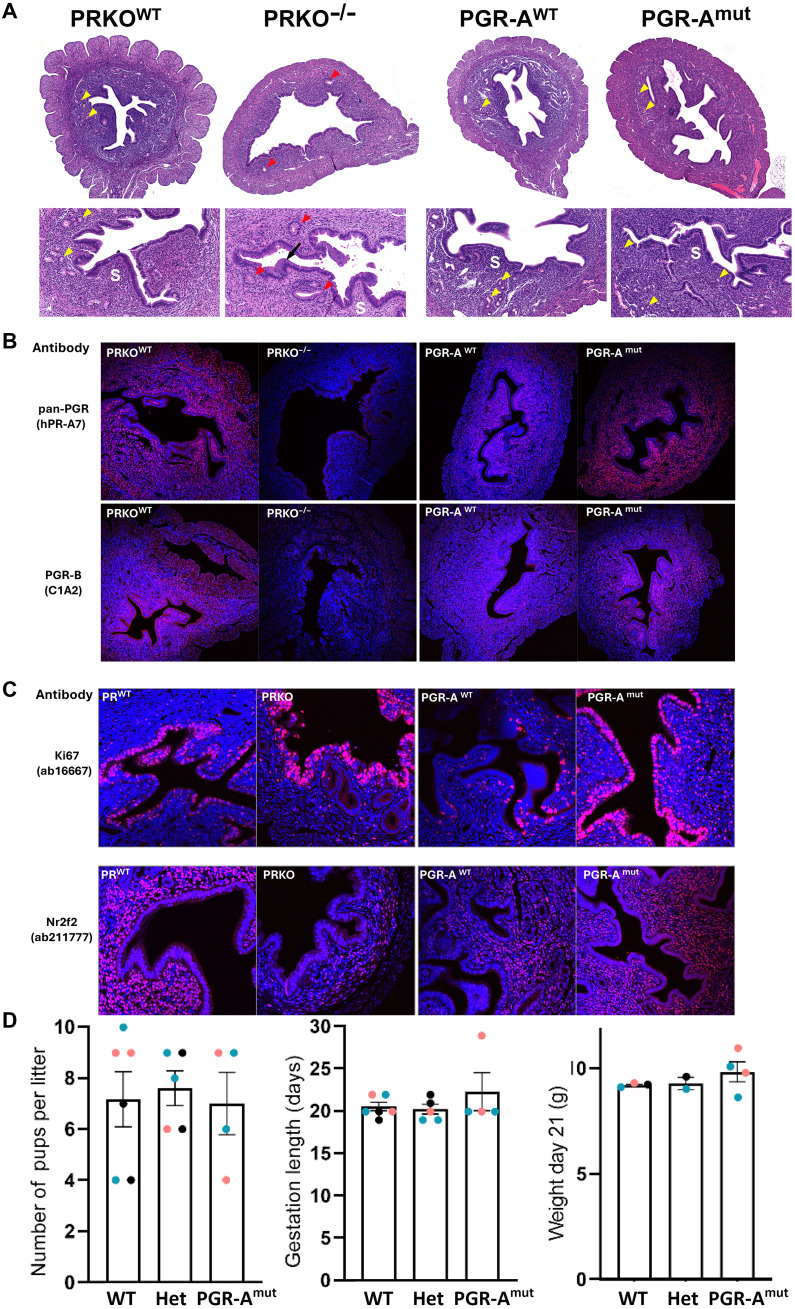
Normal uterine physiology and female fertility in PGR-A^mut^ mice. (**A**) Comparative morphology of hematoxylin and eosin–stained transverse sections of uterus from PRKO or PGR-A^mut^ lines. PRKO^−/−^ uteri show the previously reported abnormal hyperplastic luminal epithelium (black arrow), hypertrophic endometrial glands (red arrowheads), and loosely arranged stroma (S). (**B**) Immunofluorescence shows reduced total PGR (hPR-A7 antibody) and PGR-B (C1A2 antibody) in PRKO^−/−^, while total PGR and PGR-B were detected in PGR-A^mut^ uteri. (**C**) Proliferation (Ki67 antibody) and NR2F2 immunostaining in PRKO and PGR-A^mut^ uteri. (**D**) Number of pups per litter, gestation length, and offspring weight at postnatal day 21 from females of each genotype mated with WT males (*n* = 4 to 6 females per genotype with colors indicating founder line as in Fig. 3). Statistical significance was assessed by one-way ANOVA using data combined from each genotype, with *P* > 0.05 for each outcome.

While ovulation failure is the primary cause of infertility in total PRKO mice, defects in morphology of uterine luminal epithelium and glands and stroma also cause implantation failure when viable embryos are transplanted (*12*). We performed comparative assessments of adult 6- to 8-week-old PRKO and PGR-A^mut^ uteri. Morphology of PRKO uteri (Fig. 4A) replicated the previously reported phenotypes of hyperplastic luminal (LE) and hypertrophic glandular epithelia, along with disorganized and loosely arranged stromal layers (*12*). However, PGR-A^mut^ showed uterine morphology indistinguishable from their WT littermate controls (Fig 4A). Immunostaining with either pan-PGR antibody (hPR-A7) or PGR-B–specific antibody (C1A2) showed abundant PGR labeling in PRKO^WT^, PGR-A^WT^, and PGR-A^mut^ uteri, while PGR labeling was absent in PRKO^−/−^ (Fig. 4B), consistent with our Western blot observations (Fig 2). Cell proliferation assessed through immunostaining of Ki67 showed high proliferation rates in the LE of all genotypes (Fig. 4C), while the PGR-regulated gene NR2F2 (*1*, *13*) showed abundant immunofluorescent staining in the stroma, with reduced abundance in the dysmorphic stroma of PRKO^−/−^ uteri. Likewise, mammary gland morphology in adult (6 to 8 weeks old) PGR-A^mut^ females was visually normal (fig. S3).

Fertility assessments of PGR-A^mut^ females provided functional support for these morphological observations by showing overall normal physiology of the ovaries, oviducts, uteri, and mammary glands. WT, heterozygous, and homozygous PGR-A^mut^ females at 2 to 3 months of age were paired with WT males, and the number and timing of pups born and growth rates of each litter were recorded. Pregnancy was observed for six of six WT, five of six heterozygous, and four of six homozygous mutant females with 100% of pregnancies resulting in live births for all genotypes. The three nonpregnant females, two of which were PGR-A homozygous mutants, exhibited mating plugs, but no pregnancy ensued. For females that did conceive, the number of live pups born was comparable across all genotypes and in each of the three founder lines. The mean gestation length was equivalent in all genotypes. These results indicate that, in addition to normal ovulation rates in the PGR-A^mut^ mice, decidualization, implantation, maintenance of pregnancy, and parturition all occurred normally. Mammary function and lactation were also assessed as functionally normal due to visible milk in the stomachs of all newborns, and pup weights at 3 days (not shown) and 21 days of age (Fig. 3C) were not significantly different between genotypes. Together, these data demonstrate that PGR-A isoform is not essential for female fertility, with PGR-B being sufficient to mediate ovulation, uterine function, pregnancy, mammary gland development, and lactation.

## DISCUSSION

Our findings evoke a profoundly different understanding of PGR-A function in reproduction. We demonstrate that the reported PRAKO mouse model (*9*) has a frameshift mutation, leading to a truncated null product of both PGR isoforms, instead of the intended selective removal of PGR-A. This changes the interpretation of previous reports of the anovulatory infertility and defective uterine phenotypes in the PRAKO mice (*1*, *9*, *11*). Our newly generated mouse model, with confirmed in-frame mutation of the PGR-A, but not PGR-B, translation start site, contradicts the previously understood phenotype and now demonstrates that PGR-A is not essential for ovulation or any other major reproductive events necessary for female fertility. This provides evidence to substantially revise our current understanding of PGR-A isoform–specific function, and a new valid PGR-A–specific mutant model has been developed to reopen the field to understand PGR isoform functional dynamics.

The PRAKO model was first reported over 20 years ago and reported to express PGR-B normally (*9*). One subsequent study confirmed PGR-B detection by Western blot in the medial basal hypothalamus of PRAKO mice (*14*). We found that the unintended frameshift mutation was present, and PGR-B was undetectable in PRAKO mice tested from breeding pairs throughout our colony and in the founders purchased from Jackson Laboratories, indicating that the mutation did not arise de novo in our breeding colony. It is possible the aberrant mutation arose during breeding of the mice after those original studies. The original reported anovulation phenotype (*9*) was not replicated in our new PGR-A^mut^ model, which would suggest that the unintentional frameshifted null mutation may explain the similar phenotypes reported for PRKO and PRAKO mutants. Further, the loss of both PGR isoforms is most likely responsible for the reported phenotypes in PRAKO mice. A number of studies of the original PRAKO mutants have described ovarian and uterine phenotypes (*9*, *14*–*17*) that together have led to the conclusion that PGR-A has a dominant role in regulating ovulation and establishment of pregnancy. Meanwhile, it has been observed that there are minimal differences between PRAKO and total PRKO ovarian phenotypes or gene expression (*11*). The data presented here ultimately overturn this previous understanding of distinct tissue-specific functions of PGR isoform biology. Our new verified PGR-A–specific mutant mice showed normal ovulation and fertility and ovarian gene expression, as did the verified PGR-B–specific mutant mice in both ours and previously reported studies (*10*, *11*). Together, the data show that, while PGR is required for ovulation, either PGR-A or PGR-B isoform is equally sufficient for ovulation, normal maintenance of pregnancy, and parturition. PGR-B–specific mutants also revealed a specific role in mammary gland morphogenesis consistent with physiologically relevant additional coactivator interactions in this context (*10*).

Molecular mechanistic studies reproducibly show that PGR-B has significantly greater transactivation capacity (*18*, *19*), and the two isoforms interact with different coregulators (*20*, *21*), which results in a larger set of PGR-B–regulated genes (*22*, *23*). While other approaches to investigate PGR isoform functions also support nonredundant actions (*24*), our finding that the mutation of either PGR-A or PGR-B in vivo each resulted in no marked changes in ovary or uterine morphology or significant gene expression changes in granulosa cells indicates that the functional differences between isoforms have limited discernible physiological consequences.

The two alternatively translated PGR isoforms arose in early amniotes, having been identified in birds (*25*, *26*) and reptiles (*27*), and have persisted throughout mammalian evolution. In many eutherian mammals, the two isoforms play significantly different roles in pregnancy and parturition, where the myometrium switches from supporting pregnancy to promoting contraction and parturition (*23*, *28*). These divergent physiological roles are largely attributed to alternatively controlled expression of the isoforms in different cell types through DNA methylation of promoters. It is noteworthy that the mutant mice with either PGR isoform disrupted have no discernible change in uterine morphology, pregnancy duration, or parturition capability. This may be due to species differences in progesterone levels between mouse and human pregnancy at term. In mice, progesterone declines at parturition, while, in humans, there is no change in serum progesterone; thus, the PGR isoform switch may not be as important to trigger parturition in mice as in the human. While expression of exogenous PGR-B, but not PGR-A, altered parturition in mice (*23*), this sheds light on the role of PGR-B in myometrial contractility but may not reflect a physiological role of PGR isoforms in mouse parturition. It is clear that PGR is important for ovulation in humans based on antagonist treatments (*4*). We and others (*29*) have shown that both PGR-A and PGR-B are induced with equivalent transient temporal kinetics in human granulosa cells. However, no studies have investigated specific roles of the different isoforms in human ovulation. Future studies should use the improving isoform-specific detection methods to confirm spatial-temporal expression of PGR-A and PGR-B in human granulosa cells. Further, CRISPR-editing techniques similar to those used here, but in human primary granulosa cells, could determine whether downstream transcriptomes are conserved in human ovulation.

In conclusion, we found that—despite molecular evidence supporting functional differences between PGR isoforms, including higher PGR-B transcriptional activity, little overlap between cofactor interactions, and divergent regulated gene sets—the function of each individual PGR isoform from the perspective of gross physiology is largely redundant. A new PGR-A–specific mutant mouse model confirms a phenotype that is indistinguishable from WT mice as also reported for PGR-B–specific mutants. This new PGR-A mutant model may prove useful for future investigations to discover the differential functions of the PGR isoforms in nonreproductive tissues or in disease contexts such as breast cancer.

## MATERIALS AND METHODS

### Animals

The total PGR null strain, referred to here as PRKO, is the Pgr^tm1Bwo^ targeted mutation strain. The strain with intended PGR-A isoform–specific null mutation, referred to as PRAKO mice, is the strain designated Pgr^tm1Omc^. The PGR-B–specific null mutant strain, referred to as PRBKO, is the designated Pgr^tm2Omc^. Breeding pairs for these transgenic strains were initially obtained from the Jackson Laboratory, with breeding colonies established and maintained at the Laboratory Animal Services (University of Adelaide). All mice were housed in 12-hour light:12-hour dark conditions with rodent chow and water provided ad libitum. All experiments were approved by the University of Adelaide Animal Ethics Committee (approval numbers m/2018/100, m/2018/122, M-2021-075, and M-2021-093) and conducted in accordance with the National Health and Medical Research Council’s *Australian Code of Practice for the Care and Use of Animals for Scientific Purposes* and approved by the University of Adelaide Institutional Biosafety Committee (IBC dealings 14797, 14798, and 14799).

### Genotyping of PRKO, PRAKO, and PRBKO mouse strains

All offspring were genotyped twice using DNA extracted from ear biopsies collected on day 10 of age, and tail tips were collected after humane killing at the time of the experiment. For PRKO genotyping, PCR was performed with a forward primer targeting the PGR gene sequence (PGR sense: GAG GTG GAA GAG GAC AGT GG) and reverse primers targeting either the neomycin cassette (antisense null: GCC AGA GGC CAC TTG TGT AG) or the PGR sequence (antisense WT: TGG GCA CAT GGA TGA AAT CCA CC). These primers amplify the mutant allele DNA to produce a 200–base pair (bp) product or the WT allele to produce a 262-bp product. The 20-μl PCR reaction contained 1× Goflexi Reaction Buffer (Promega, Madison, WI, USA), 3 mM MgCl_2_, 5% dimethyl sulfoxide, 0.25 mM deoxynucleotide triphosphates (dNTP), 0.5 μM each primer, and 0.5 U of Goflexi Taq Polymerase (Promega), made up in sterile H_2_O, and, then, 1 μl of genomic DNA was added. DNA was amplified by thermocycler: 5 min at 95°C, 35 cycles × (1 min at 95°C, 30 s at 60°C, and 30 at 72°C), 5 min at 72°C, and hold at 4°C. PCR products were resolved on a 2% agarose gel with 0.1% GelRed stain and visualized on GelDoc Imager (Bio-Rad, Hercules, CA, USA).

PRAKO and PRBKO ATG start site mutations were genotyped using PCR to amplify PGR in the region of interest, followed by TaqMan fluorescent probe assay (Thermo Fisher Scientific, Waltham, MA, USA) using Fluorescein Amidite (FAM) fluorescent dye–tagged DNA probes targeting either mutation or VIC fluorescent dye–tagged WT sequence. Each 10-μl reaction contained 5 μl of ProAmp TaqMan MasterMix, 0.25 μl of TaqMan assay PGR-A or PGR-B–specific probes, 3.75 μl of sterile water, and 1 μl of genomic DNA or water for the negative control. The QuantStudio12K Flex System (Thermo Fisher Scientific) was used under the following conditions: 30 s at 60°C, 10 min at 95°C, 40 cycles × (15 s at 95°C and 1 min at 60°C), 30 s at 60°C, and then hold at 4°C. The ratio of VIC(WT):FAM(KO) fluorescence is plotted on an allelic discrimination plot, in which WT mice have high WT:KO ratio, KO mice have low WT:KO ratio, and heterozygous animals have similar levels of both fluorescent signals.

PRAKO primers:

1) PGR amplification sense: GCC ATC ACT TCC TGG TGT CT

2) PGR amplification antisense: TGG GTG GTG ACA GTC CTT TG

3) WT probe: (VIC) CCC GCT CAT GAG TCG GC

4) Mutant probe: (FAM) CCC GCT CGC TAG TCG GC

PRBKO primers:

1) PGR amplification sense: CTC CTC CCA GGA GAC AGG

2) PGR amplification antisense: GAA CCT GCG GAT CCT TTG C

3) WT probe: (VIC) TTG GGT CGT CAT GAC TG

4) Mutant probe: (FAM) TTG GGT CGT CCT GAC TG

### Generation of PGR-A–specific null mice by CRISPR editing

South Australian Genome Editing performed CRISPR editing, in which zygotes were collected from C57BL/6 females 1 day after mating and microinjected with Cas9 mRNA (100 ng/μl), pegRNA (50 ng/μl; **TGTTGTCCCCGCTCATGAGT**gtttcagagctagaaatagcaagttgaaataaggctagtccgttatcaacttgaaaaagtggcaccgagtcggtgc**GATCTCTGGCCGgCTAGCGAGCGGGGACAAC**, spacer and reverse transcriptase template are in bold), and a single-stranded DNA template [50 ng/μl; 5′-CCAGAGCTTCCAGAAGACCCCCGCAGTGTCCCTGCTACCAAAGGGTTGTTGTCCCCGCTCGCTAGcCGGCCAGAGATCAAGGCTGGCGACAGCTCCGGGACAGGAGCAGGACAGAAGGTGCTGCCC-3′ designed to substitute the PGR-A–specific start codon (amino acid 166) for alanine (ATG>GCT)]. A silent mutation was introduced at serine-167 (AGT>AGC) to introduce a NheI restriction site for genotype screening. A pegRNA was used instead of a standard gRNA as this sequence displayed high cleavage activity in pilot Prime Nuclease experiments. CRISPR-edited zygotes were transferred (20 to 21 per oviduct) to pseudopregnant Swiss dams, and resultant litters were genotyped by PCR using forward primer (5′-tctctggggtagaagccact-3′) and reverse primer (5′-gggtggtgacagtcctttgg-3′) with cycling conditions: 2 min at 95°C, 35 cycles × (30 s at 95°C, 30 s at 60°C, and 30 s at 72°C), and 5 min at 72°C. NheI digest of amplified DNA was used to identify successful recombination. Individual males with positive NheI digest were subjected to Sanger sequencing to confirm the intended gene edit with no additional sequence changes. Three males with the exact intended mutation were selected to initiate three individual founder lines. This newly developed strain is designated PGR^tm1Dlr^. Each founder male was crossed with a WT C57BL6/J female, and resultant pups were assessed for genotype. In each case, five pups were sequenced and confirmed consistent inheritance of the correct allele. Subsequently, heterozygous F_1_ siblings were mated (without crossing founder lines) for individual testing of the three lines.

PGR^tm1Dlr^ animals were genotyped using TaqMan single-nucleotide polymorphism fluorescent probe assay (Thermo Fisher Scientific) with the following primers:

1) Probe 1 VIC (WT): CCCGCTCATGAGTCGGC

2) Probe 2 FAM (KO): CCCGCTCGCTAGCCGGC

3) Forward: GCCATCACTTCCTGGTGTCT

4) Reverse: TGGGTGGTGACAGTCCTTTG

### Ovulation assessment

Female mice were injected intraperitoneally with 5 IU of equine chorionic gonadotropin (eCG) in 100 μl of saline and, then, after 44 to 46 hours, with 5 IU of hCG in 100 μl of saline to stimulate ovulation. Sixteen hours post-hCG, mice were culled by cervical dislocation, and ovaries and attached oviducts were collected directly into warmed alpha minimum essential medium (αMEM). Under a dissecting light microscope, oviducts were torn with fine forceps to release the COCs, and the COC number was counted by two different researchers blind to genotypes. Isolated ovaries were fixed in ice cold 4% paraformaldehyde for histology.

### Histology and immunofluorescence

Fixed ovaries and uteri were embedded in paraffin wax and sectioned at 5 μm (Leica Microsystems Pty Ltd., Macquarie Park, Australia) and then adhered onto glass slides. Hematoxylin and eosin–stained sections were imaged at ×40 resolution on the NanoZoomer Digital Slide Scanner (Hamamatsu Photonics, Hamamatsu City, Japan). For immunofluorescence, slides were dewaxed with xylene and then rehydrated. Antigen retrieval was performed using citrate buffer [pH 6; 9 ml of 0.1 M citric acid and 41 ml of 0.1 M sodium citrate made up to 1 liter of H_2_O (pH6)]. After blocking in 1% bovine serum albumin/9% normal goat serum, anti-PGR (1:500; hPRa7, Thermo Fisher Scientific; D8Q2J, Cell Signaling Technology), anti–PGR-B (1:500; C1A2, Cell Signaling Technology), anti-Ki67 or anti Nr2f2 (1:500; Abcam), or equivalent concentration of matching immunoglobulin G (IgG) isotype control (rabbit IgG or mouse IgG; Cell Signaling Technology, Danvers, MA, USA) was applied at 4°C overnight. Slides were washed and incubated with secondary antibodies (1:2000) and Hoechst33343 (1:500; H1399, Life Technologies, Carlsbad, CA, USA) for 1 hour at room temperature (RT). After washing, sections were covered using Pertex mounting medium (Medite, Burgdorf, Germany) and coverslips (1.5 μm, ProSciTech, Kirwan, Australia) and imaged using an Olympus FV3000 confocal microscope (Olympus, Tokyo, Japan).

Mammary gland whole mounts were prepared by spreading dissected mammary gland #4 on glass slides. After air drying, slides were submerged in Carnoys solution for 24 hours and then in carmine alum [0.2% carmine dye and 0.5% aluminum potassium sulfate (Sigma-Aldrich)]. After washing in 70% ethanol (0.024 N HCl), slides were dehydrated and mounted in DPX mounting medium (Sigma-Aldrich) and then imaged on Zeiss AxioScan 7.

### Western blot

Lysates were prepared by homogenizing tissue in cold radioimmunoprecipitation assay buffer (Millipore, Billerica, MA, USA) and 1% protease inhibitor cocktail (Sigma-Aldrich, St. Louis, MI, USA). Supernatant was cleared by centrifuge at 8000*g* for 10 min and transferred to a new tube. Equal protein concentrations were denatured in Lithium Dodecyl Sulfate (LDS) buffer and 1:100 β-mercaptoethanol and 25 to 40 μg of protein run on 4 to 12% precast acrylamide gels (Invitrogen, Waltham, MA, USA) in 1× Bolt MES running buffer (Invitrogen) at 165 V for ∼1 hour. Electrophoresed protein was either transferred to nitrocellulose and blocked with Odyssey blocking buffer (blots in Fig. 1C) or transferred to polyvinylidene difluoride membrane and blocked with 5% skim milk in tris-buffered saline–0.1% Tween 20 (all other Western blots). Membranes were incubated shaking with primary antibody for 50 to 60 min at RT, then washed three times for 5 min in 0.1% Tween buffer. Fluorescent or horseradish peroxidase (HRP) secondary antibody (1:10,000) was applied for 1 hour at RT in darkness, before washing three times for 5 min. For chemiluminescent detection of HRP secondary antibodies (1:10,000), SuperSignal West Atto Ultimate Sensitivity Substrate (Thermo Fisher Scientific) was applied for 1 min and then blot imaged using the chemiluminescence imaging on the ChemiDoc MP imaging system (Bio-Rad). Fluorescent secondaries were scanned using the LiCor Odyssey 9120 Imaging System (Li-Cor, Lincoln, LE, USA). The following primary antibodies were used: anti-PGR (1:500; D8Q2J, Cell Signaling Technology), anti-PGR-B (1:1000; C1A2, Cell Signaling Technology), anti–β-actin (1:5000; A1978, Sigma-Aldrich), and anti–histone-3 (1:1000; 9715S, Cell Signaling Technology).

### Fertility assessment

Two- to 3-month-old PGR-A^Mut^ females from each founder line were paired with heterozygous males from their respective founder lines. Each male was mated to three different females (of mixed genotype), with at least 4 days between each pairing. The breeding pairs were maintained until a copulatory plug was observed or for a maximum of 6 days. The gestation length was calculated as the time between mating setup to the date of birth. The pups were counted at birth and weighed at 3 and 21 days of age, and weight were averaged across the litter to calculate average pup weight.

### RNA sequencing

Female PGR-A WT and KO mice at 21 to 27 days old were hormonally stimulated with 5 IU of eCG for 46 hours, followed by 5 IU of hCG, and were humanely killed at 8 hours post-hCG. Granulosa cells were collected from ovaries by follicle puncturing and snap-frozen in liquid nitrogen. A total of four biological replicates per genotype were obtained, each consisting of granulosa cells pooled from two to three mice. RNA was extracted with the Monarch Total RNA Miniprep Kit (New England Biolabs), including deoxyribonuclease treatment, as per the manufacturer’s instructions. RNA quality was assessed using the RNA ScreenTape system (Agilent). mRNA library was prepared using the Illumina TruSeq Stranded mRNA kit (Illumina). Paired-end sequencing was performed on the NovaSeq 6000 S1 sequencing system (Illumina). Overrepresented adapters were checked using FastQC, and AdapterRemoval was used to trim when required. Reads were aligned to the GENCODE mouse transcriptome (mm10), and transcripts were quantified using Salmon. Transcript-per-million–normalized count was calculated, and differential expressed genes were assessed using limma-edgeR R packages. Differential gene expression was defined as log fold change of ≥1 (fold change of ≥±2) and a Benjamini-Hochberg adjusted *P* value of ≤0.01.
