# Supplementary material for: Reassessing the role of progesterone receptor isoforms PGR-A and PGR-B in female fertility
Source: Sci Adv. 2026 Aug 12;12(33):eadz7757. doi: 10.1126/sciadv.adz7757 (PMC13464633; doi:10.1126/sciadv.adz7757)
Supplement: Supplementary file 1 [file sciadv.adz7757_sm.pdf]

Science Advances
AAAS

Supplementary Materials for

Reassessing the role of progesterone receptor isoforms PGR-A and PGR-B in female fertility

Doan T. Dinh *et al.*

Corresponding author: Darryl L. Russell, darryl.russell@adelaide.edu.au

Sci. Adv. **12**, eadz7757 (2026)
DOI: 10.1126/sciadv.adz7757

This PDF file includes:

Figs. S1 to S3

Dinh et al. Reassessing the role of Progesterone Receptor isoforms PGR-A and PGR-B in female fertility

Supplementary Figure 1

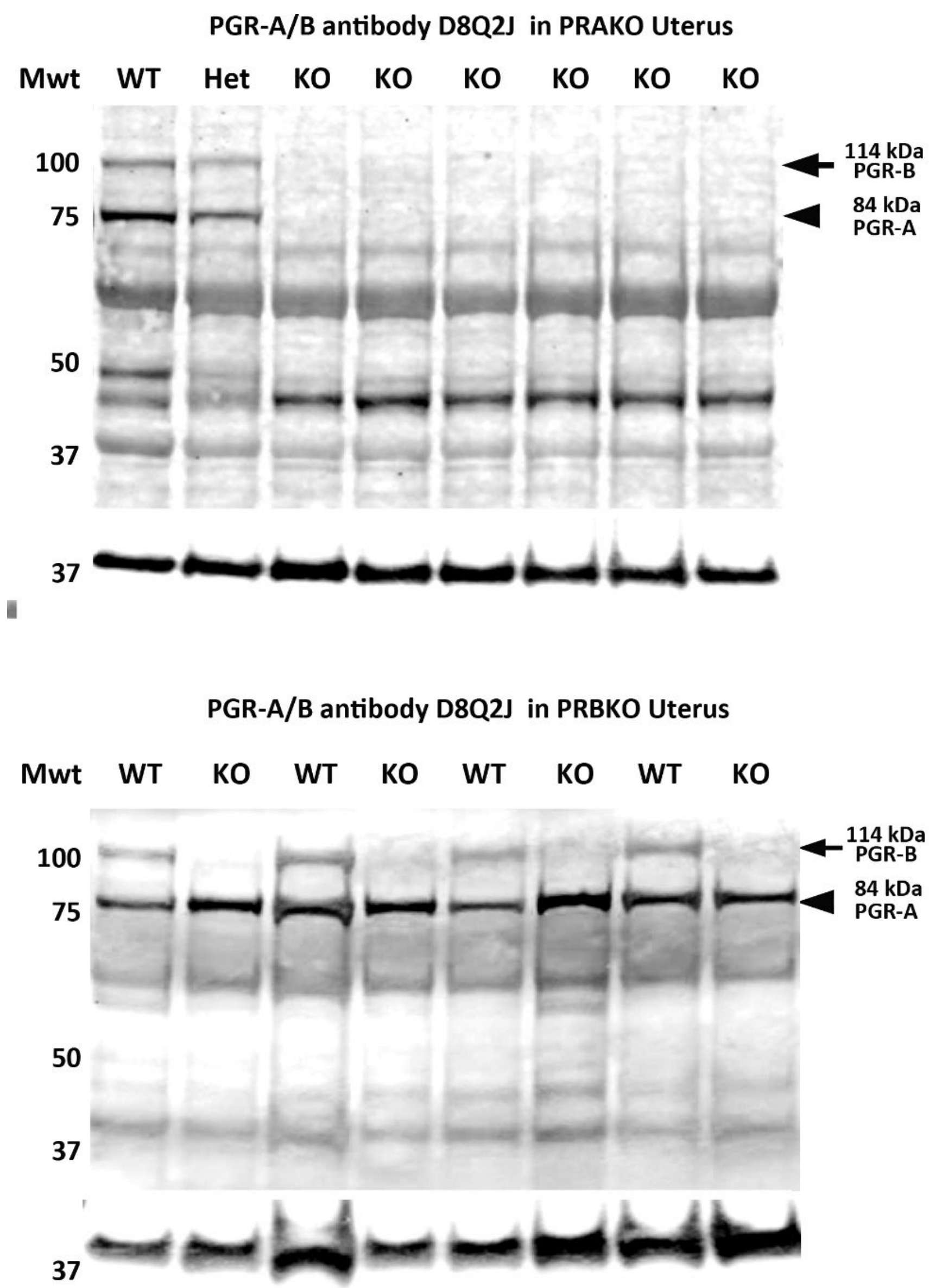

Supplementary Figure 1. Western blots of uterus extracts in wild type (WT), heterozygous (Het) or knockout (KO) from PRAKO or PRBKO mice using the total PGR antibody D8Q2J. Lower blots show 42 kDa β-actin loading control (antibody A1978, Sigma-Aldrich, 1:5000).

Supplementary Figure 2

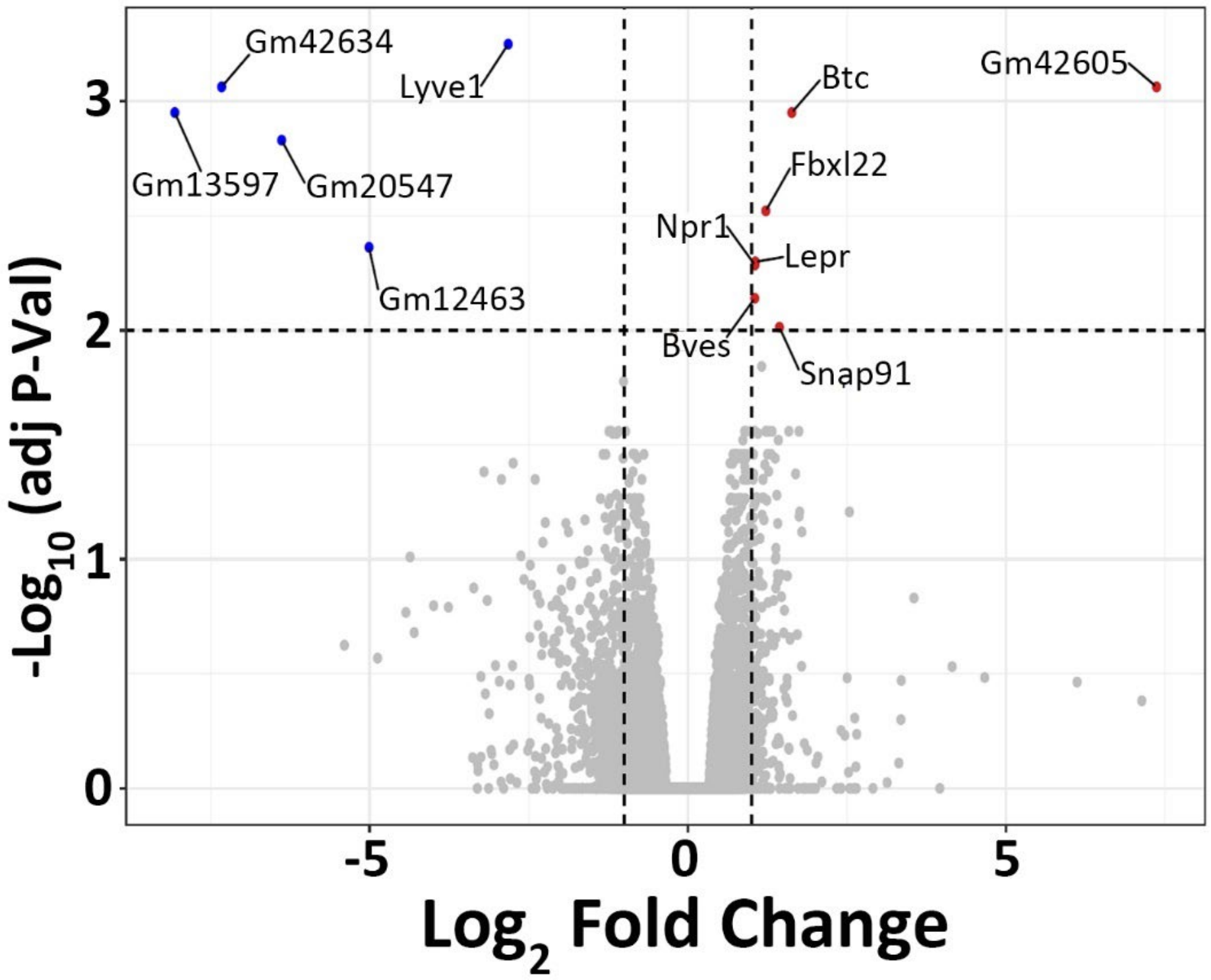

Supplementary Figure 2. Volcano plot of differential gene expression in RNA-seq analysis of WT versus PGR-A^{mut} granulosa cells after 8 h hCG treatment.

Supplementary Figure 3

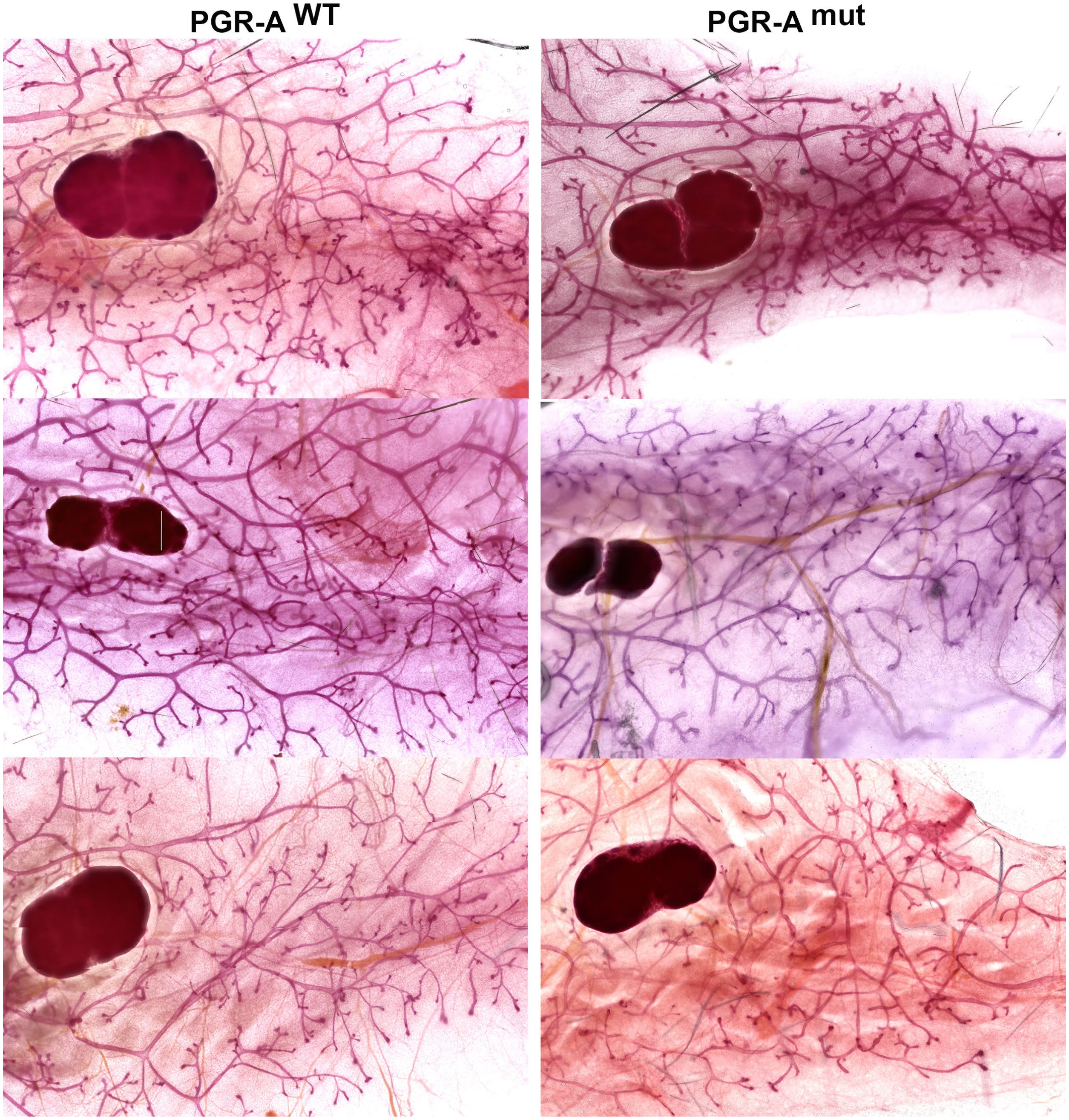

Supplementary Figure 3. Mammary gland morphology in Carmine alum–stained whole mount of the #4 abdominal mammary glands from three individual adult PGR-A^{WT} or PGR-A^{mut} mice
